# Infection of Goose with Genotype VIId Newcastle Disease Virus of Goose Origin Elicits Strong Immune Responses at Early Stage

**DOI:** 10.3389/fmicb.2016.01587

**Published:** 2016-10-04

**Authors:** Qianqian Xu, Yuqiu Chen, Wenjun Zhao, Tingting Zhang, Chenggang Liu, Tianming Qi, Zongxi Han, Yuhao Shao, Deying Ma, Shengwang Liu

**Affiliations:** ^1^College of Animal Science and Technology, Northeast Agricultural UniversityHarbin, China; ^2^Division of Avian Infectious Diseases, State Key Laboratory of Veterinary Biotechnology, Harbin Veterinary Research Institute, Chinese Academy of Agricultural SciencesHarbin, China

**Keywords:** NDV, goose, AvBD, TLR, cytokines, iNOS

## Abstract

Newcastle disease (ND), caused by virulent strains of Newcastle disease virus (NDV), is a highly contagious disease of birds that is responsible for heavy economic losses for the poultry industry worldwide. However, little is known about host-virus interactions in waterfowl, goose. In this study, we aim to characterize the host immune response in goose, based on the previous reports on the host response to NDV in chickens. Here, we evaluated viral replication and mRNA expression of 27 immune-related genes in 10 tissues of geese challenged with a genotype VIId NDV strain of goose origin (go/CH/LHLJ/1/06). The virus showed early replication, especially in digestive and immune tissues. The expression profiles showed up-regulation of Toll-like receptor (TLR)1–3, 5, 7, and 15, avian β-defensin (AvBD) 5–7, 10, 12, and 16, cytokines [interleukin (IL)-8, IL-18, IL-1β, and interferon-γ], inducible NO synthase (iNOS), and MHC class I in some tissues of geese in response to NDV. In contrast, NDV infection suppressed expression of AvBD1 in cecal tonsil of geese. Moreover, we observed a highly positive correlation between viral replication and host mRNA expressions of TLR1-5 and 7, AvBD4-6, 10, and 12, all the cytokines measured, MHC class I, FAS ligand, and iNOS, mainly at 72 h post-infection. Taken together, these results demonstrated that NDV infection induces strong innate immune responses and intense inflammatory responses at early stage in goose which may associate with the viral pathogenesis.

## Introduction

Newcastle disease (ND), caused by Newcastle disease virus (NDV), is regarded as one of the most important avian diseases (Häuslaigner et al., 2009). The virus caused an economically serious disease in almost all poultry (Alexander, 1988; Sun et al., 2013). Waterfowls, such as duck and goose, are generally considered to be natural reservoirs or carriers of NDV, even those most virulent for chicken (Alexander, 2001; Alexander and Senne, 2008). However, ND outbreaks in domestic waterfowl have frequently been reported in East Asian countries, including Korea, Japan, and China, since the 1980s (Liu et al., 2003; Lee et al., 2004; Mase et al., 2009). In the affected flocks of duck, egg production sharply declined by about 70%, morbidity was about 80%, and mortality varied from 30 to 50%. The diseased birds showed diarrhea and nervous signs, and the dead birds mainly manifested by focal hemorrhage, necrosis of the intestinal mucosa, and congestion and hemorrhage of the ovarian follicles (Liu et al., 2010, 2015; Dai et al., 2013). Similarly, serious ND outbreaks have also been reported in flocks of geese in China (Liu et al., 2003). The geese challenged with NDV of goose origin showed clinical signs such as anorexia, white diarrhea, depression, nasal discharges, ocular, and dead (Wan et al., 2004; Häuslaigner et al., 2009). In Asia, the main virulent NDV strain belongs to Genotype VIId, which is a major threat to the poultry industry, including goose (Hu et al., 2015). Thus, understanding the underlying mechanism of NDV pathogenesis, as well as development of novel alternative therapeutic approaches is of great importance. Recently, most reports have focused on the molecular characteristics and pathogenicity of epidemic strains (Kumar et al., 2011; Chen et al., 2012; Wang et al., 2015), while the host–virus interactions remain largely unknown.

Innate immunity is considered to be the first line of host defense against virus infection. The host innate response play an important role early in the process on an infection, as some of these responses may prevent the initial viral replication or they may send appropriate signals in order to initiate other innate mechanisms as well as adaptive responses. Prominent among these are Toll-like receptors (TLRs) (Abdul-Careem et al., 2009). In mammals, at least 13 TLRs have been identified. In chicken, TLR1A and B, TLR2A and B, TLR3, TLR4, TLR5, TLR7, TLR15, and TLR21 have been identified (Paul et al., 2013). It has been shown that several TLRs recognize viral pathogen-associated molecular patterns (PAMPs): TLR3, detects double-stranded RNA (dsRNA) derived from viral replication whereas single stranded RNA (ssRNA) are detected by TLR7 and TLR8 (Sang et al., 2008). TLRs provide various means of limiting virus replication until adaptive immune responses are activated. For example, TLR3 plays a critical role in eliminating herpes simplex virus-2 infection in mouse reproductive tract (Ashkar et al., 2004; Abdul-Careem et al., 2009), or inhibiting NDV replication in HeLa cells (Cheng et al., 2014). Upon recognize invading virus, TLRs activate downstream signaling cascades, leading to the secretion of soluble factors, such as cytokines and host defense peptides (HDPs), which in turn mediate innate immune responses to limit viral replication (Seth et al., 2006; Takeuchi and Akira, 2010).

Newcastle disease virus induced host immune responses in chickens (Marina and Hanson, 1987). NO production *in vitro* was upregulated in chicken peripheral blood mononuclear cells (PBMCs) and heterophils in response to NDV challenge (Sick et al., 2000; Ahmed et al., 2007). Accordingly, inducible NO synthase (iNOS) mRNA expression was upregulated in NDV-infected chicken PBMCs *in vitro* (Ahmed et al., 2007). In addition, NDV also induced interferon (IFN)-α and IFN-β mRNA in chicken macrophages (Sick et al., 1998), IFN-γ mRNA in PBMCs (Ahmed et al., 2007), and IFN-α, IFN-β, interleukin (IL)-1β and IL-6 in chicken splenic leukocytes (Rue et al., 2011). In agreement with the observations *in vitro*, there are multiple genes induced in spleen of NDV-infected chickens *in vivo*, including some chemokines and cytokines, types I and II IFNs, IFN effectors and iNOS (Rue et al., 2011). These findings were confirmed by our recent results that pigeon NDV induces immune responses characterized by activation of TLRs, particularly TLR3 and TLR7, avian β-defensin (AvBD) 2 and 10, and iNOS of pigeons at 7 days post infection (Li et al., 2015). Up to now, particular attentions are mostly given to the pathogenicity and origin of NDV, while studies on host immune response to NDV infection are scarce. Here, a genotype VIId of class II NDV strain of goose origin (go/CH/LHLJ/1/06) (Xu et al., accepted), which was isolated from geese in 2006 in our laboratory, was selected as the model pathogen. In order to characterize the host immune response in goose, based on the previous reports on the host response to NDV in chickens, we examined the virus replication and induction of innate host responses in tissues of geese challenged with the NDV strain in this study.

## Materials and Methods

### Ethics Statement

All animal experimental procedures were approved by the Ethical and Animal Welfare Committee of Heilongjiang Province, China (License no. SQ20150508).

### Virus and Animals

Goose isolate of NDV (go/CH/LHLJ/1/06) was isolated from the field in 2006 in Heilongjiang Province, China, and proved to be a virulent NDV strain with mean dead time of 51 h in embryonating chicken eggs, and intracerebral pathogenicity index of 1.86 (Alexander et al., 1998; Xu et al., accepted). In addition, it was found that the virulent NDV strain causes a mortality rate of 20% in geese (Xu et al., accepted).

One-day-old geese were hatched in this study. The geese were reared to observe health status for 40 days old. Clinical health of the geese was confirmed by a histopathological examination. In addition, the sera of the birds were confirmed to be negative for NDV-specific hemagglutination inhibition antibodies before experiments were done. The challenge test was conducted in isolators in biosafety level 3 facilities under negative pressure.

### Nucleotide Sequencing of Immune Molecules in Geese

The total RNA was extracted and cDNA was synthesized as previously described (Li et al., 2015). The resultant cDNA product was used for subsequent PCR by using Ex-Taq polymerase (Takara Bio, Shiga, Japan). The primers were designed by aligning the nucleotide sequences of respective genes from geese (*Anser*), chickens (*Gallus gallus*), and ducks (*Anas platyrhynchos*) using software of DNAStar (Lasergene Corp, Madison, WI, USA). Briefly, the primers of 18S rRNA, TLRs (2, 3, 4, 5, 7, and 15), AvBDs (1, 2, 3, 5, 6, 9, and 10), MHC class I, and cytokines (IL-1β, IL-2, IL-6, IL-8, IL-18, and IFN-γ), were designed based on respective nucleotide sequences of geese. The nucleotide sequences of TLR1, AvBDs (4, 7, 12, and 16), iNOS, and FAS ligand (FASLG) of both chickens (*G. gallus*) and ducks (*A. platyrhynchos*) were aligned by using software of DNAstar as well. The consensus sequences were selected and used for primer design (**Table 1**). The PCR products were cloned and sequenced as previously described (Li et al., 2015). The resultant plasmids were used as respect control for subsequent quantitative RT-PCR.

**Table 1:** PCR primer sequences and predicted product lengths.

Target mRNA	Sense primer (5′–3′)	Antisense primer (5′–3′)	Product size (bp)	GenBank accession no.
AvBD4 (RT-PCR)	5′-ATCGTGCTCCTCTTTGTGGCAGTTCA-3′	5′-CTACAACCATCTACAGCAAGAATACT-3′	171	–
AvBD7 (RT-PCR)	5′-GGAGACAGAAGGCAGCGGTGAT -3′	5′-GGAGACAGAAGGCAGCGGTGAT -3′	326	KR018386
AvBD12 (RT-PCR)	5′-CAGCCCTGCTGCTCYCCAGCAG -3′	5′-TCAGGTCTTGGTGGGAGTTGGTGACAGAGG	230	KR018387
		TTTACAGCAGAGAATGAC -3′
AvBD16 (RT-PCR)	5′-ATGAAGATCCTCTGCCTGCTCTTC -3′	5′-TTAGTCGTACACAGTCCG(T)GCAGCACGGGAC -3′	180	-
AvBD1 (real time RT-PCR)	5′-GAAACAAGGAGAAATGTCATCG -3′	5′-ATGGGGGTTGTTTCCAGGAGC -3′	183	JQ359443
AvBD2 (real time RT-PCR)	5′-GATTGTCTTCGCCCCAGCGGGA -3′	5′-TTATACATCCCATGGCGATTTG-3′	137	HQ909024
AvBD3 (real time RT-PCR)	5′-GAACTGCCACTCAGTGCAGAAT -3′	5′-ATGGGGGTTGTTTCCAGGAGC-3′	182	NM_204650
AvBD4 (real time RT-PCR)	5′-ATCGTGCTCCTCTTTGTGGCAGTTCA-3′	5′-CTACAACCATCTACAGCAAGAATACT-3′	171	-
AvBD5 (real time RT-PCR)	5′-GCTGTCCCTTCCTCGAGGATC-3′	5′-GGAATACCATCGGCTCCGGC-3′	139	HM452159
AvBD6 (real time RT-PCR)	5′-GTCAGCCCTACTTTTCCAGC -3′	5′-GCCCACCTGTTCCTCACAC -3′	142	JQ359442
AvBD7 (real time RT-PCR)	5′-CCAAGGAGTGGCAGGTCAG -3′	5′-CCTCGCACAGCAGGAACC -3′	142	KR018386
AvBD9 (real time RT-PCR)	5′-GCTTACAGCCAAGGAGATGCT-3′	5′-GGAGCTAGGTGCCCATTTGCA-3′	144	HQ909023
AvBD10 (real time RT-PCR)	5′-GGTTCTGCCGATGCGCCTTTTG-3′	5′-CTGCGCCGGAATCTTGGCAC-3′	147	HQ909025
AvBD12 (real time RT-PCR)	5′-TCACGGGCACGCACACG-3′	5′-GAGAATGACGGGCTCGAAGC-3′	115	KR018387
AvBD16 (real time RT-PCR)	5′-TGGAATCAGAGGTGGAGTTTG-3′	5′-GCAGCACGGGACGATAAG-3′	88	-
TLR1 (real time RT-PCR)	5′-AGTCCATCTTTGTGTTGTCGCC-3′	5′-ATTGGCTCCAGCAAGATCAGG-3′	127	JF823982
TLR2 (real time RT-PCR)	5′-GATTGTGGATAACATCATCGACTC-3′	5′-AGAGCTGCTTTCAAGTTTTCCC-3′	294	JN982474.1
TLR3 (real time RT-PCR)	5′-TCAGTACATTTGCAACACTC-3′	5′-AGCATCATAATCATACCCTTCC-3′	256	KC292270.1
TLR4 (real time RT-PCR)	5′-AATCTGAAATCACTGAGCTCAAAT-3′	5′-GAGATGTTAAACCATAGAAG-3′	190	HQ436371.1
TLR5 (real time RT-PCR)	5′-CCTTGTGCTTTGAGGAACGAGA-3′	5′-CACCCATCTTTGAGAAACTGTC-3′	124	JQ711137.1
TLR7 (real time RT-PCR)	5′-TTCTGGCCACAGATGTGACG-3′	5′-CCTTCAACTTCGCAGTGCAG-3′	219	JQ910168.1
TLR15 (real time RT-PCR)	5′-GTTCTCTCTCCCAGTTTTGTAAACAGC-3′	5′-AACGTTCATTTGTTGTTTTTAGGAC-3′	262	JQ014619.1
iNOS (real time RT-PCR)	5′- GAACAGCCAGCTCATCCGATA -3′	5′- CCCAAGCTCAATGCACAACTT -3′	103	U34045
18s (real time RT-PCR)	5′-TCCCAGTAAGCGCGAGTCAT-3′	5′-ACGGGCGGTGTGTACAAAG-3′	64	AB064942
IL-1β(real time RT-PCR)	5′-CCGCAAAGTGAGGCTCAACA-3′	5′-GTAGCCTTTGATGCCCAGC-3′	100	JF505290
IL-2 (real time RT-PCR)	5′-ACCGAGAGCTGACCAACTTT-3′	5′-ATCACCCACACTAAGAGCAT-3′	177	AY392557.1
IL-6 (real time RT-PCR)	5′-AGATGGTGATAAATCCTGATGA-3′	5′-CGGTTTTCTCCATAAATGAAGT-3′	150	HQ436372
IL-8 (real time RT-PCR)	5′-ATGAACGGCAAACTTGGGGCT-3′	5′-GCCAGAATTGCCTTTACGATCAG-3′	278	AB213393
IL-18 (real time RT-PCR)	5′-TGAAATCTGGCAGCGGAATGAAC -3′	5′-TCCCATGTTCTTCCTCACAACA-3′	135	JF 505289
IFN-γ (real time RT-PCR)	5′-GCCACACATCAAAAACCTGTCT-3′	5′-GGAGACTGGCTCCTTTTCCTT-3′	207	EU030377
MHC class I (real time RT-PCR)	5′-AAGAAGGAGAAGGGCTACAA-3′	5′-GACATGCAGATGCAATTATGC-3′	207	AY387653.1
FASLG (real time RT-PCR)	5′-TAACAGGAAACCCCACACAGC-3′	5′-CCGGAAGAGCACATTGGAGTA-3′	149	NM_001031559
18S rRNA (real time RT-PCR)	5′-TCC CAG TAA GCG CGA GTC AT-3′	5′-ACG GGC GGT GTG TAC AAA G-3′	65	AB064942

### Bioinformatic Analysis

Basic searches were conducted with BLAST program analysis^1^. Phylogenetic tree was performed from aligned amino acid sequences by the neighbor-joining method with 1,000 bootstraps using the MEGA4 program software (Molecular Evolutionary Genetics Analysis, version 6.0, Armonk, NY, USA).

### Experimental Design and Real-Time RT-PCR of mRNAs in Tissues

At the age of 40 days, the geese were allotted randomly to three groups. Groups 1 and 2 have 10 birds each and was inoculated intranasally with 100 μl of the NDV strain (go/CH/LHLJ/1/06) at 10^6^ 50% egg infective doses. Group 3 has twenty birds and was inoculated with 100 μl of phosphate-buffered saline only, and served as a control. At 36 and 72 h post infection (hpi), five geese from Groups 1 and 3 were killed and ten tissue samples, including brain, trachea, lungs, kidneys, liver, proventriculus, spleen, cecal tonsil, Harderian glands, and bursa of Fabricius were collected. All of these collected tissues were used for real-time RT-PCR analysis of NDV and host genes. Birds in group 2 and the remaining birds in group 3 were kept to observe the clinical signs and mortality for 3 weeks post-challenge. The dead birds were examined for the gross lesions in different organs.

One-step Real-time PrimeScript RT-PCR (Takara Biotechnology, Dalian, China) was used to evaluate the mRNA levels of the selected genes as described previously (Li et al., 2015). The preparation of the real-time RT-PCR followed the QIME requirement http://www.clinchem.org/content/55/4/611.long. Briefly, RNA was extracted as described above. The assays were performed using 2 μL of total RNA and the One-step Real-time PrimeScript^®^ RT-PCR kit (Takara Biotechnology, Dalian Co., Ltd.) in a 20-μL reaction on a LightCycler^®^ 480II Real-Time PCR system (Roche, Basel, Switzerland) according to previous studies (Li et al., 2015; Xu et al., 2015). Serial tenfold dilution of plasmids containing goose18S rRNA, TLRs (1, 2, 3, 5, 7, and 15), AvBDs (1–7, 9, 10, 12, and 16), cytokines (IL-1β, IL-6, IL-8, and IL-18), iNOS, MHC class I and FASLG were used as controls. The mRNA of these genes was evaluated. All amplifications were conducted in triplicate. The concentration of target cDNA in a sample was deduced from the crossing point obtained and from the corresponding standard curve. The data are expressed for each sample as the copy number of each target cDNA normalized to that of the reference gene (18S rRNA), as described previously (Li et al., 2015).

One-step Real-time PrimeScript RT-PCR was also used for detecting the viral RNA of the NDV strain in the tissues of geese as described previously (Guo et al., 2014). The primers and probe were designed based on the *M* gene sequence of the NDV strain used in this study. The primers and probe were as follows:

forward, 5′–CTCAGTGATGTGCTCGGACC–3′;
reverse, 5′–CCTGGGGAGAGGCATTTGCTA–3′;
probe, 5′–[FAM]TTCTCTAGCAGTGGGACAGCCTGC[BHQ1]–3′ BH.

### Statistical Analysis

Data are expressed as means ± SD. The statistical significance was assessed by using SAS software (1996) as previously described (Li et al., 2015). Correlation between the relative gene expression of immune molecules and NDV was performed using Pearson’s tau using SAS software (1996), and *P* < 0.05 was considered to be statistically significant.

The nucleotide sequences of both of anser_AvBD7 and anser_AvBD12 obtained in current study are available from GenBank under the accession numbers KR018386 (anser_AvBD7) and KR018387 (anser_AvBD12). The nucleotide sequences of anser_AvBD4, anser_AvBD16, TLR1, FASLG and iNOS are shown in detail in Supplementary Figure S2–S6 in the Supplementary Materials, due to nucleotide sequences of them are shorter than 200 bp.

## Results

### Sequence Analysis of Immune Molecules

**FIGURE 1:**
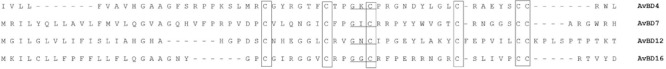
**Deduced amino acid sequence alignment of four novel avian β-defensin (AvBDs) from geese.** The six conserved cysteines (C) are framed. The GXC motif is underlined. Dashes indicate that no identical or conserved residues were observed.

Using a set of primers designed to amplify conserved AvBDs sequences, four novel AvBDs (4, 7, 12, and 16) were identified from both spleen and bone marrow of healthy geese. The open reading frames (ORFs) of two of these novel AvBDs contained 201 and 198 nt, respectively, and encoded 66 and 65 amino acids, respectively. The other two contained only parts of the ORFs with 171 and 180 bp, and encoded 56 and 59 amino acids, respectively. A BLASTN search revealed that the sequence of the first peptide (56 amino acids) showed the highest amino acid identity (80.9%) to chicken AvBD4, comparing with other AvBDs and defensins of the mammals. Hence, the first peptide was designed as goose AvBD4. The sequence of the remaining peptides (66, 65, and 59 amino acids) shared the highest amino acid identities to chicken AvBD7 (84.4%), AvBD12 (87.4%) and duck AvBD16 (66.3%), respectively, and designed as goose AvBD7, AvBD12, and AvBD16, respectively. Moreover, the GXC motif and the six cysteine residues were found in the predicted amino acid sequences of these four peptides that are conserved across all β-defensins. These four novel AvBDs were named anser_AvBD4, anser_AvBD7, anser_AvBD12, and anser_AvBD16 (**Figure 1**). Phylogenetic analyses were performed on the amino acid sequences of β-defensins, including these four novel AvBDs, the other reported AvBDs, and some mammalian β-defensins (Supplementary Figure S1). All these β-defensins segregated into eight distinct clades. Anser_AvBD4 formed a branch with AvBD4 from other avian species; anser_AvBD7 formed a branch with AvBD7 and AvBD6 from other avian species; anser_AvBD12 formed a branch with AvBD12–14 from chickens and two mammalian β-defensins (*Mus musculus* Defb2 and *Sus scrofa* PBD2); and anser_AvBD16 formed a branch with AvBD3, 8, 16, 103a, and 103b from other avian species.

Other than the former four novel AvBDs, we tried to amplify other AvBDs (i.e., 8, 11, 13, and 14), which have not been characterized from geese till now. Unfortunately, none of these AvBDs were identified in the current study. In addition, partial sequences of 18S rRNA, TLRs (1, 2, 3, 5, 7, and 15), AvBDs (1–3, 5, 6, 9, and 10), cytokines (IL-1β, IL-2, IL-6, IL-8, IL-18, and IFN-γ), iNOS, MHC class I, and FASLG were amplified using respective primers (**Table 1**) from both spleen and bone marrow of healthy geese. The sequences of these peptides shared >95% identity to the respective sequences from geese that were available in a public database (data not shown).

### Pathogenic Observation and Viral Replication in Geese

The geese in the control group did not show any clinical signs and none of these geese died during the experiment. In contrast, two geese died in group 2 on 3 dpi although none of the NDV-challenged geese showed clinical observations. Gross lesions, such as hemorrhage and edema in the proventriculus, hemorrhagic changes in the trachea, congestion in the lung, slight enlargement and congestion in livers were observed in the dead birds (Xu et al., accepted).

**FIGURE 2:**
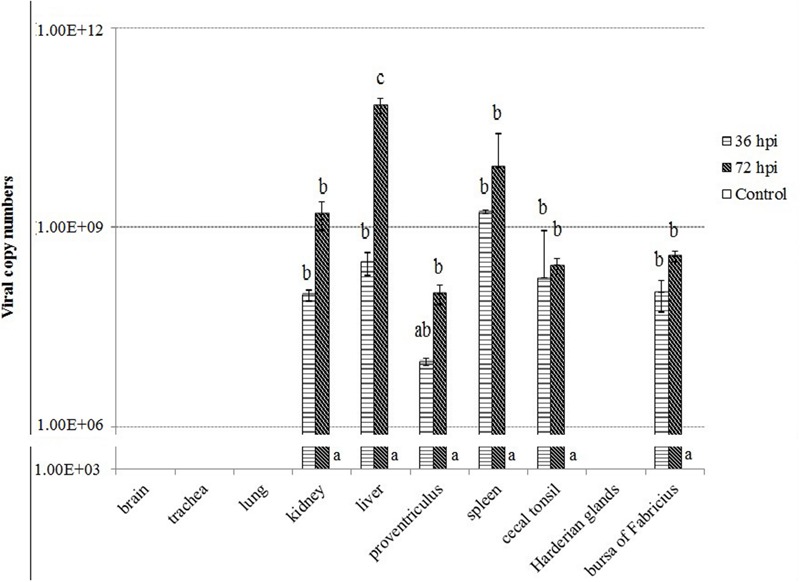
**Viral RNA copies in the tissues of geese in response to Newcastle disease virus (NDV) infection.** Viral RNA copy numbers in the tissue samples from five geese in each group were measured by quantitative PCR at 36 and 72 hpi. The control is the mean of results of Control-36 h and Control-72 h, due to results from both groups are almost the same. All assays were performed in triplicate, with five replicates per experiment, and each bar is the mean ± SD. ^a,b,c^The values with different letters are significantly different (*P* < 0.05).

To understand the severity of pathology in the early infection period, we examined virus replication by real-time RT-PCR in ten tissues of geese of the control, 36 and 72 hpi, including brain, trachea, lungs, kidneys, liver, proventriculus, spleen, cecal tonsil, Harderian glands, and bursa of Fabricius. Samples from the control birds were negative. In contrast, the virus showed early replication, and was detected as early as 36 hpi, increased by 72 hpi in six tissues (i.e., kidneys, liver, proventriculus, spleen, cecal tonsil, and bursa of Fabricius). In addition, the virus replication was not detectable in brain, trachea, lung, and Harderian glands of infected geese (**Figure 2**).

### Upregulation of TLR Expression in Response to NDV Infection

**FIGURE 3:**
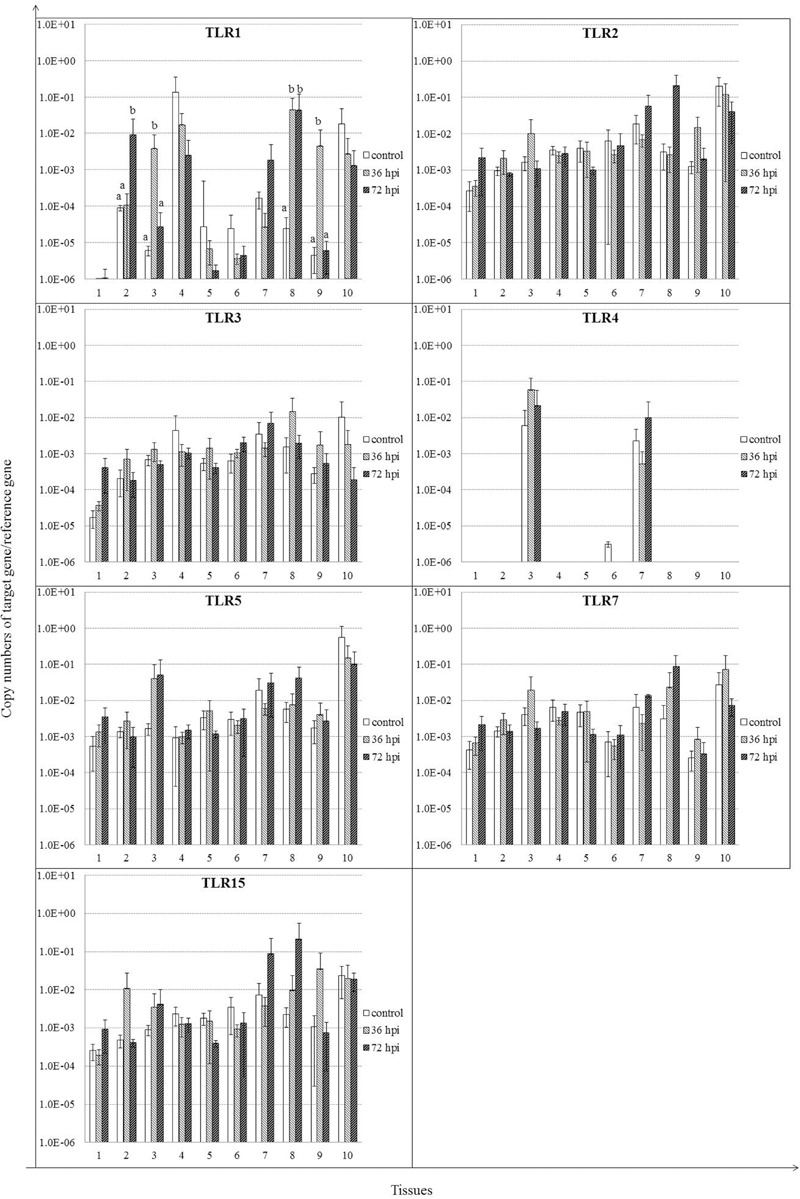
**Relative gene expression of toll-like receptors (TLRs) in the tissues of geese in response to NDV infection.** (1) Brain, (2) Trachea, (3) lung, (4) kidneys, (5) Liver, (6) proventriculus, (7) spleen, (8) cecal tonsil, (9) Harderian gland, (10) bursa of Fabricius. cDNA copy numbers in the tissue samples from five geese of each group were measured by quantitative PCR at 36 and 72 hpi. TLR levels were normalized to the levels of 18S rRNA in the same samples. The control is the mean of results of Control-36 h and Control-72 h, due to results from both groups are almost the same. All assays were performed in triplicate, with five replicates per experiment, and each bar is the mean ± SD. ^a,b^The values with different letters are significantly different (*P* < 0.05).

We analyzed expression patterns of TLR1–5, 7, and 15 of geese in response to NDV infection (**Figure 3**). The majority of these genes showed upregulation in infected geese. It is notable that TLR1 mRNA expression levels were upregulated significantly in several tissues of infected geese, including trachea by 72 hpi, lung by 36 hpi, cecal tonsil for the duration of the experiment, and Harderian glands by 36 hpi (*P* < 0.05). Despite lack of significant difference, expression of the other TLRs was also upregulated in most tissues of infected geese, including: TLR2 in cecal tonsil by72 hpi; TLR3 in brain by 72 hpi; TLR5 in lung by 36 and 72 hpi; TLR7 in cecal tonsil by 36 and 72 hpi; and TLR15 in trachea and Harderian glands by 36 hpi, and cecal tonsil by 72 hpi (*P* > 0.05). In contrast to extensive distribution of the above TLRs, TLR4 was detected only in lungs, proventriculus, and spleen, and no obvious difference was found between infected and control geese.

### Differential Expression of AvBDs in NDV-Infected Geese

**FIGURE 4:**
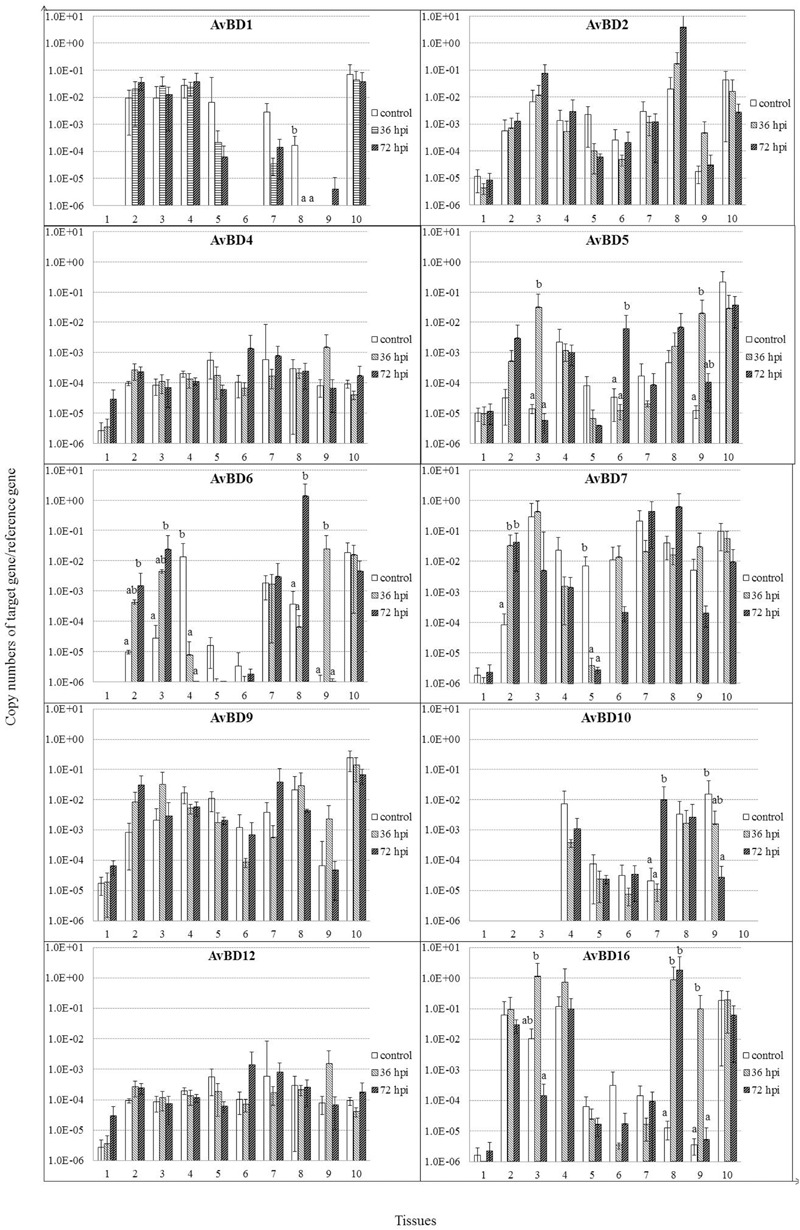
**Relative gene expression of AvBDs in the tissues of geese in response to NDV infection.** (1) Brain, (2) Trachea, (3) lung, (4) kidneys, (5) Liver, (6) proventriculus, (7) spleen, (8) cecal tonsil, (9) Harderian gland, (10) bursa of Fabricius. cDNA copy numbers in the tissue samples from five geese of each group were measured by quantitative PCR at 36 and 72 hpi. AvBD levels were normalized to the levels of 18S rRNA in the same samples. The control is the mean of results of Control-36 h and Control-72 h, due to results from both groups are almost the same. All assays were performed in triplicate, with five replicates per experiment, and each bar is the mean ± SD. ^a,b^The values with different letters are significantly different (*P* < 0.05).

Most of the AvBDs measured were detectable in all the tissues from both the control and NDV-infected geese, except for AvBD1, 3 6, and 10 (**Figure 4**). AvBD3 was not detected in these tissues from either infected or control geese (data not shown). AvBD1 expression was completely suppressed in cecal tonsil by 36 and 72 hpi (*P* < 0.05). In the NDV-infected geese, there was significant upregulation in mRNA expression of AvBD5 in several tissues, including lung by 36 hpi, proventriculus by 72 hpi, and Harderian glands by 36 hpi, compared to the controls (*P* < 0.05). Expression of AvBD6, 7, 10, and 16 exhibited variable regulation in some tissues of geese in response to NDV infection. AvBD6 was significantly upregulated in trachea, lung, and cecal tonsil by 72 hpi, and in Harderian glands by 36 hpi (*P* < 0.05). In kidney, its expression was decreased by 36 hpi, and completely suppressed by 72 hpi (*P* < 0.05). AvBD7 was upregulated in trachea, but suppressed in liver for the duration of the experiment (*P* < 0.05). Furthermore, AvBD10 expression was upregulated in spleen by 72 hpi, but suppressed in Harderian glands by 72 hpi (*P* < 0.05). AvBD16 expression was increased by 36 hpi, but suppressed by 72 hpi in lung (*P* < 0.05). In addition, AvBD16 expression was upregulated in cecal tonsil for the duration of the experiment, and in Harderian glands by 36 hpi, compared to the controls (*P* < 0.05). In contrast to variable regulation of the above AvBDs in response to NDV infection, there was no significant regulation of AvBD2, AvBD4, and AvBD9 in all these tissues (*P* > 0.05).

### NDV Infection Induces Cytokine Responses

**FIGURE 5:**
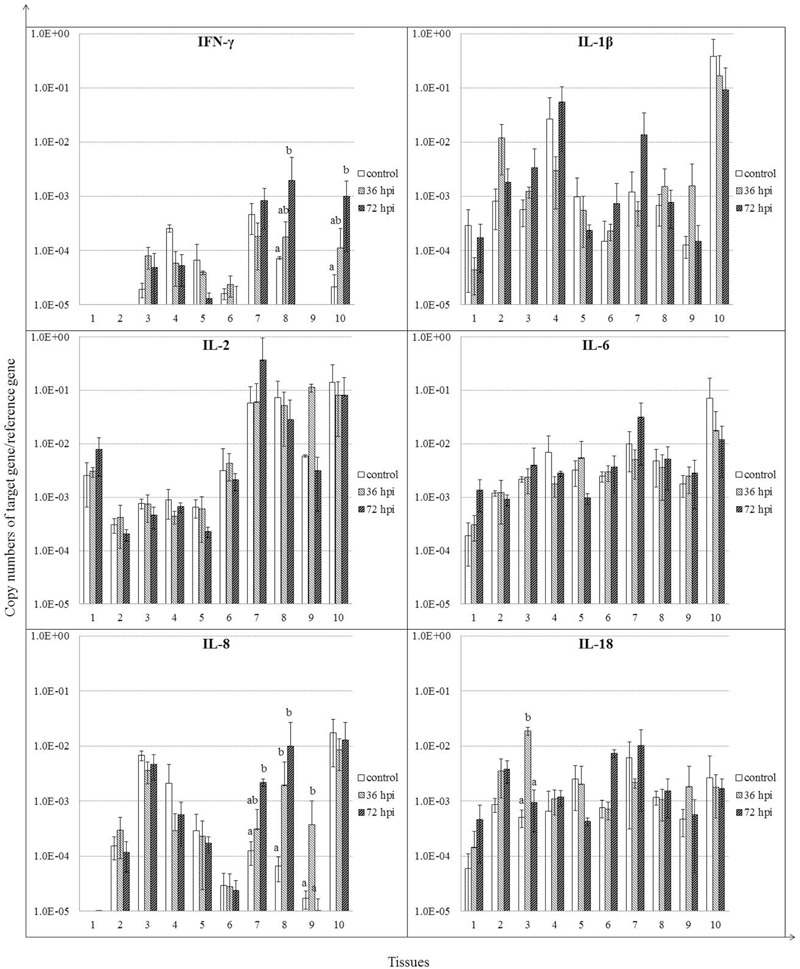
**Relative gene expression of cytokines in the tissues of geese in response to NDV infection.** (1) Brain, (2) Trachea, (3) lung, (4) kidneys, (5) Liver, (6) proventriculus, (7) spleen, (8) cecal tonsil, (9) Harderian gland, (10) bursa of Fabricius. cDNA copy numbers in the tissue samples from five geese of each group were measured by quantitative PCR at 36 and 72 hpi. Cytokine levels were normalized to the levels of 18S rRNA in the same samples. The control is the mean of results of Control-36 h and Control-72 h, due to results from both groups are almost the same. All assays were performed in triplicate, with five replicates per experiment, and each bar is the mean ± SD. ^a,b^The values with different letters are significantly different (*P* < 0.05).

We analyzed the expression patterns of IFN-γ and selected inflammatory cytokines (**Figure 5**). IFN-γ mRNA was induced by 36 hpi (*P* > 0.05), continued to increase by 72 hpi (*P* < 0.05) in both cecal tonsil and bursa of Fabricius. Expression of IL-8 was induced, although insignificantly by 36 hpi, and continued to rise by 72 hpi in spleen and cecal tonsil (*P* < 0.05). IL-8 mRNA was also induced in Harderian glands by 36 hpi (*P* < 0.05). Furthermore, IL-18 expression was increased significantly only in lung by 36 hpi (*P* < 0.05). For IL-1β, IL-2, and IL-6, little significant difference was detected at each time point in all tissues between the control and NDV-infected geese (*P* > 0.05).

### Expression of iNOS in NDV-Infected Geese

**FIGURE 6:**
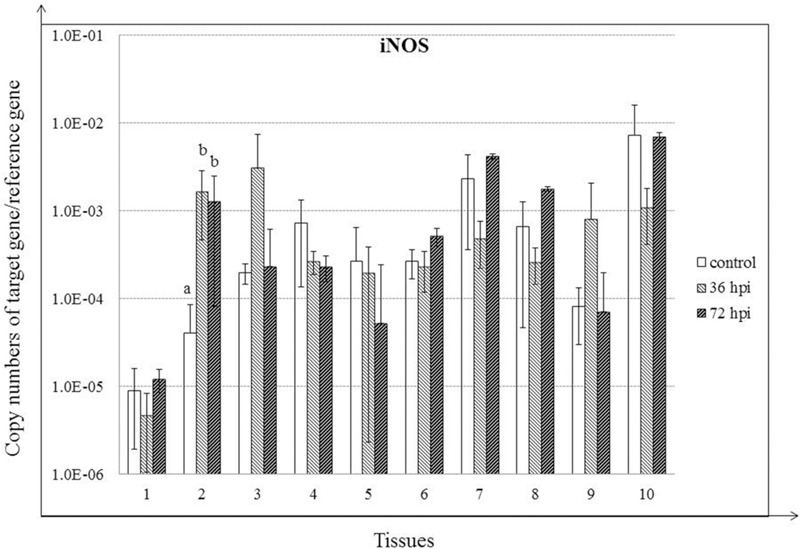
**Relative gene expression of iNOS in the tissues of geese in response to NDV infection.** (1) Brain, (2) Trachea, (3) lung, (4) kidneys, (5) Liver, (6) proventriculus, (7) spleen, (8) cecal tonsil, (9) Harderian gland, (10) bursa of Fabricius. cDNA copy numbers in the tissue samples from five geese of each group were measured by quantitative PCR at 36 and 72 hpi. iNOS levels were normalized to the levels of 18S rRNA in the same samples. The control is the mean of results of Control-36 h and Control-72 h, due to results from both groups are almost the same. All assays were performed in triplicate, with five replicates per experiment, and each bar is the mean ± SD. ^a,b^The values with different letters are significantly different (*P* < 0.05).

Expression of iNOS was increased significantly only in trachea for the duration of the experiment (*P* < 0.05). In addition, despite the lack of significant differences, iNOS expression was also increased in lung and Harderian glands by 36 hpi (*P* > 0.05) (**Figure 6**).

### Expression of MHC Class I and FASLG in Response to NDV Infection

**FIGURE 7:**
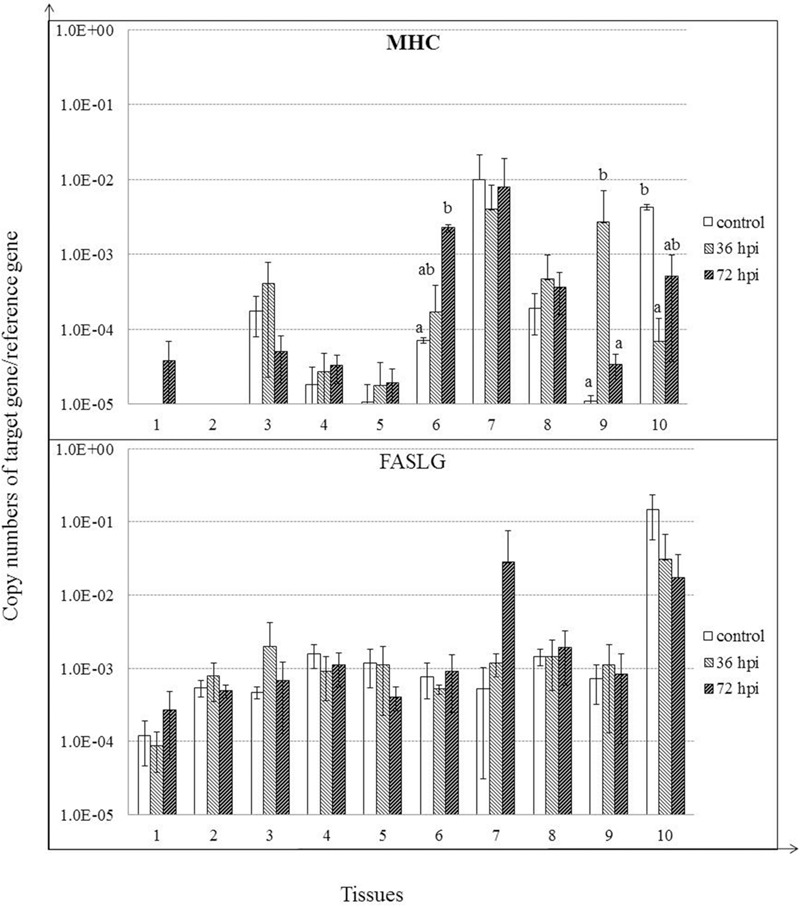
**Relative gene expression of MHC class I, and FASLG in the tissues of geese in response to NDV infection.** (1) Brain, (2) Trachea, (3) lung, (4) kidneys, (5) Liver, (6) proventriculus, (7) spleen, (8) cecal tonsil, (9) Harderian gland, (10) bursa of Fabricius. cDNA copy numbers in the tissue samples from five geese of each group were measured by quantitative PCR at 36 and 72 hpi. Gene levels were normalized to the levels of 18S rRNA in the same samples. The control is the mean of results of Control-36 h and Control-72 h, due to results from both groups are almost the same. All assays were performed in triplicate, with five replicates per experiment, and each bar is the mean ± SD. ^a,b^The values with different letters are significantly different (*P* < 0.05).

MHC class I expression was induced, although insignificantly by 36 hpi (*P* > 0.05), and continued to rise by 72 hpi in proventriculus (*P* < 0.05). Furthermore, MHC class I was significantly induced in Harderian glands by 36 hpi (*P* < 0.05). In contrast, MHC class I expression was suppressed in bursa of Fabricius by 36 hpi (*P* < 0.05) and 72 hpi (*P* > 0.05). For FASLG, despite the lack of significant regulation, expression of FASLG was clearly induced in spleen by 72 hpi (*P* > 0.05) (**Figure 7**).

### Relationship between Gene Expression of NDV and Immune Molecules in Spleen

**FIGURE 8:**
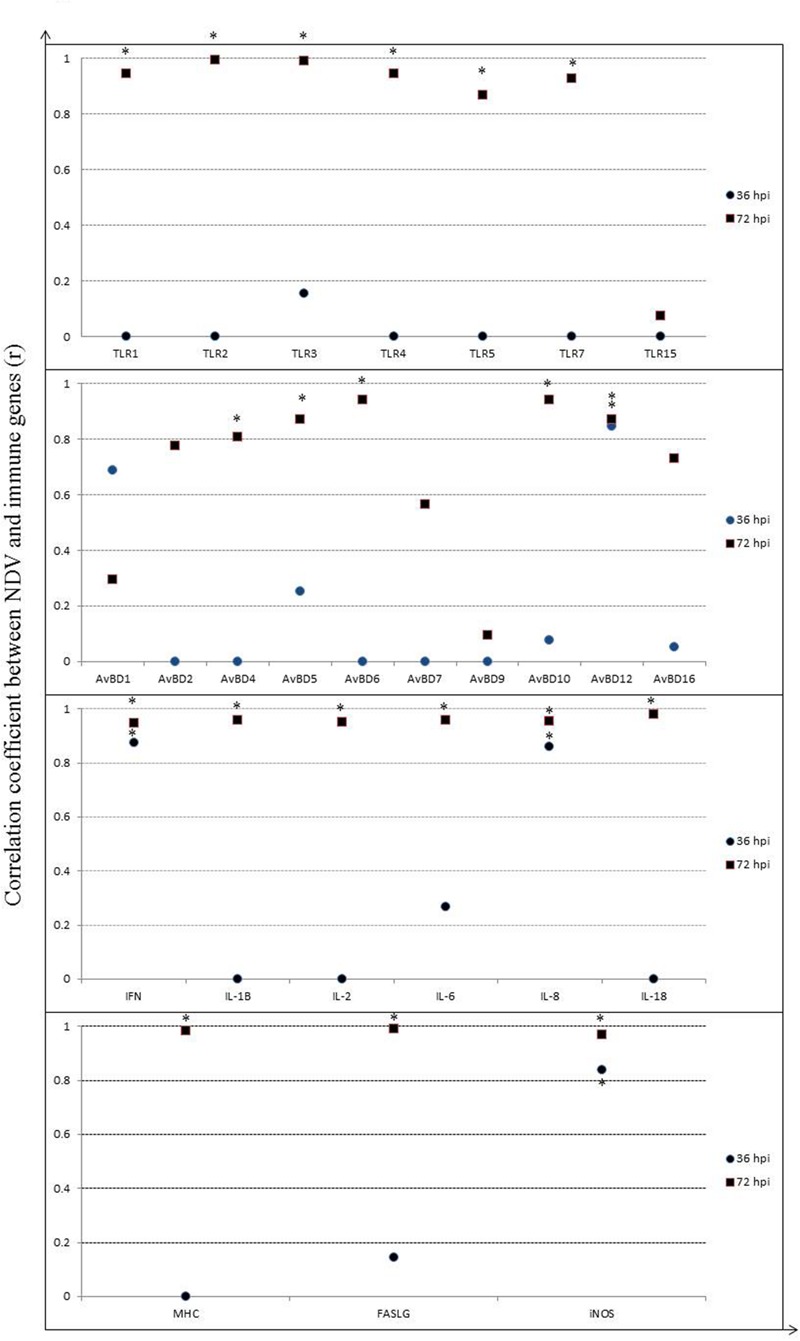
**Correlation between viral RNA copies and relative gene expression of host immune molecules in spleen of geese in response to NDV infection.** Significant correlation is indicated by a star (^∗^).

Induction of host immune-related genes was accompanied by NDV replication in several tissues but their responses likely varied at each time point post-infection. Therefore, to clarify the relationship between viral replication and host immune response to NDV infection, we assessed the correlation between viral replication and gene expression in the spleen after infection, considering the importance of the spleen for both innate and adaptive immune responses (**Figure 8**). A significant positive correlation was shown between viral replication and mRNA expression for all TLRs measured by 72 hpi (*P* < 0.05), except for TLR15, but negative or few correlations by 36 hpi (*P* > 0.05). In addition, it is demonstrated that a high positive correlation was showed between viral replication and mRNA expression for TLR7 (*P* < 0.05). For AvBDs, significant positive correlation was observed only between viral replication and AvBD12 expression by 36 hpi (*P* < 0.05), whereas, we observed a significantly positive correlation between viral replication and expression of AvBD4, 5, 6, 10, and 12 by 72 hpi (*P* < 0.05) (**Figure 8**). Similarly, for cytokines, there was a high positive correlation between viral replication and mRNA expression for IFN-γ and IL-8 by 36 hpi (*P* < 0.05), while mRNA expression for all the cytokines had a high positive correlation with viral replication by 72 hpi (*P* < 0.05). Furthermore, mRNA expression of MHC class I and FASLG showed low correlation with viral replication by 36 hpi (*P* > 0.05), but high positive correlation with viral replication by 72 hpi (*P* < 0.05). iNOS expression showed a highly positive correlation with viral replication at both time points post-infection (*P* < 0.05). The results suggest that NDV infection caused an active host immune response mainly by 72 hpi.

## Discussion

In this study, viral RNA was detectable in kidneys, liver, proventriculus, spleen, cecal tonsil, Harderian glands, and bursa of Fabricius as early as 36 hpi, and increased at 72 hpi. Furthermore, high viral load was found in both liver and spleen of NDV-infected geese at each time point. In contrast, high level of viral replication was detected in all of these 10 tissues measured in dead geese (died on 3 dpi) (Xu et al., accepted). Surprisingly, no apparent respiratory signs were observed in all of the NDV-infected geese although 20% of the birds were died due to NDV infection (Xu et al., accepted). This was different from that of chickens which showed obvious respiratory signs after NDV infection (Ecco et al., 2011). In addition, gross lesions were also observed in various organs in dead goose, such as hemorrhage and edema in the proventriculus and slight enlargement and congestion in liver. Interestingly, no obvious gross lesions were observed in the trachea and lung of the live birds, in contrast to hemorrhagic changes in the trachea and congestion in the lung and brain of dead birds (Xu et al., accepted). These results suggested differences between chicken and goose after NDV infection.

The expression levels of 27 immune-related genes in 10 tissues of geese infected with the NDV strain were analyzed in the present study. The actual mechanisms responsible for host defense against viral replication are still not known. However, it is likely that TLRs play a part initially. TLRs have a role to activate the innate immunity by recognizing PAMPs, in mammals as well as birds (Hghihghi et al., 2010; Li et al., 2015). To date, TLRs have been identified in several avian species, such as duck, chicken, goose, and pigeon. Most TLRs have a potential role in antiviral responses, regardless of species (Hghihghi et al., 2010; Ma et al., 2012a, 2013; Li et al., 2015; Xu et al., 2015). In the current study, expression of TLR1–5, 7, and 15 was evaluated in NDV-infected geese, and most of these TLRs, except TLR4, were induced in different tissues. Consistent with the current results, recent evidence revealed that both TLR3 and TLR7 are induced by NDV in chickens, as well as by PPMV-1, a variant strain of NDV, in pigeons (Rue et al., 2011; Cheng et al., 2014; Li et al., 2015). It has also been demonstrated that overexpression of TLR3 enhances activity of IFN-β promoter and transcription factor nuclear factor-κB, thereby decreasing viral protein synthesis and titer (Cheng et al., 2014). These results strongly suggest that TLR1–3, 5, 7, and 15 actively participate in the recognition of the innate proinflammatory response after NDV infection.

An innate host response can be induced by the interaction between TLRs and their specific ligands, leading to the secretion of HDPs and cytokines (Abdel-Mageed et al., 2014). It is well established that defensins are the important components of host early innate immunity beyond cytokines (Ma et al., 2012a,b, 2013; Cuperus et al., 2013; Li et al., 2015; Xu et al., 2015). In poultry, only the β-defensins are present (Lynn et al., 2007). So far, more than 50 AvBDs have been characterized in different bird species (Cuperus et al., 2013). In recent studies, AvBD1–3, 5, 6, 9, and 10 have been isolated from geese (Ma et al., 2012b, 2013). Although initially described primarily as antibacterial agents, recent studies have also demonstrated direct antiviral potential (Ma et al., 2011, 2012a; Li et al., 2015; Xu et al., 2015). In addition to the former seven AvBDs, four novel AvBDs (4, 7, 12, and 16) were identified from geese in the present study. We found that expression of all of these AvBDs showed variable regulation in some tissues in geese in response to NDV infection. AvBD1 expression was suppressed at an early stage in cecal tonsil of geese. In contrast, AvBD1 expression was unchanged in tissues of chickens challenged with infectious bronchitis virus (Xu et al., 2015), or increased in tissues of ducks infected with duck hepatitis virus (Ma et al., 2012a). These findings suggest that effect of viruses on expression of AvBD1 depends upon the organs examined, the breed of birds, or viral strains. Furthermore, consistent with previous studies (Ma et al., 2011, 2012a), expressions of AvBD5 and AvBD12 was upregulated in several tissues of geese in response to NDV infection. Regulation of AvBD6, 7, 10, and 16 expressions varied among tissues in geese in response to NDV challenge in this study. It was upregulated in some tissues but suppressed in others. These findings were partly consistent with previous studies on birds in response to other viral infections (Ma et al., 2011, 2012a; Li et al., 2015; Xu et al., 2015). To our surprise, a highly positive correlation was observed between viral replication and expressions of AvBD4–6, 9, 10, 12, and 16 in spleen at various time points post-infection; AvBDs have been shown to possess direct antiviral activity against viruses *in vitro* (Ma et al., 2011, 2012a; Li et al., 2015; Xu et al., 2015). However, the actual mechanisms responsible for this observation require further investigation.

In this study, we selected IFN-γ, IL-1β, IL-8, IL-2, IL-6, and IL-18 as indicators of antiviral and proinflammatory responses. These cytokines have been studied to understand the host immune response to a wide range of avian viruses and are important in infection control and virus clearance in birds (Ecco et al., 2011; Rue et al., 2011; Lee et al., 2013; Rasoli et al., 2014; Guan et al., 2015; Kang et al., 2015; Chimeno et al., 2016). The cytokine gene analysis showed that NDV infection in geese increased expressions of IFN-γ, IL-8, and IL-18. In addition, we also found the increased iNOS expression in NDV-infected geese. Moreover, the expression of these molecules highly correlates to the viral replication in the spleen. This result was similar to other reports which showed the correlation between high level of virus replication and intense inflammatory response caused by genotype VIId NDV in chickens and ducks (Ecco et al., 2011; Rue et al., 2011; Rasoli et al., 2014; Hu et al., 2015; Kang et al., 2015). The present findings are also in agreement with the observations in chickens infected by other avian viruses, including avian influenza virus (Lee et al., 2013; Guan et al., 2015), infectious bursal disease virus (Chimeno et al., 2016), and laryngotracheitis virus (Vagnozzi et al., 2016). Taken together, these results demonstrated that NDV infection induced strong innate immune responses and intense inflammatory responses at early stage in goose. These responses may associate with the viral pathogenesis.

Interestingly, MHC class I was significantly induced in proventriculus by 72 hpi and in Harderian glands by 36 hpi, whereas its expression was significantly suppressed in bursa of Fabricius by 36 hpi (*P* < 0.05). While, FASLG expression was slightly induced only in the kidneys and spleen, and remained at the basal level in the other tissues of geese in response to the NDV infection. The present result is inconsistent with that reported by Sarmento et al. (2008) in chickens infected by avian influenza H5N1 viruses. It is also found that expression of MHC class I was upregulated by avian influenza H3N2 virus (Tong et al., 2004). The possible reason might be that highly pathogenic viruses, such as avian influenza H5N1virus, have a mechanism to inhibit the expression of MHC class I. During infection with low pathogenic viruses such as avian influenza H3N2, as well as the current NDV strain, the viruses may be sensed by dendritic cells or macrophages and internalized into phagosomes. Here, they undergo proteolytic processing to produce antigenic peptides delivered by MHC-I molecules to the cell surface (Luo et al., 2014).

To our surprise, despite no viral replication detected in trachea and lung of infected geese at both time points, significant differences were found on expressions of several molecules, including TLRs (3, 5, and 15), AvBDs (5–7), IL-18, and iNOS in either of both tissues between the control and infected goose. Interestingly, result from our recent study showed viral RNA (go/CH/LHLJ/1/06) could be detected in both trachea and lung tissues in dead geese on 3 dpi (Xu et al., accepted). The result implies that host early defensing response against viral infection is active in both of trachea and lung, although the viral replication is later in both tissues than in the other tissues. However, the actual mechanisms responsible for the observation are not known and need to further study.

## Conclusion

The present study demonstrated that NDV infection induces strong innate immune responses and intense inflammatory responses at early stage in goose which may associate with the viral pathogenesis. This study is the analysis on the host early immune response to challenge with virulent NDV and further investigation are required to characterize how NDV affects differential host responses of geese.

## Author Contributions

QX, YC, WZ, TQ, and TZ performed the experiments. CL performed the calculation. ZH and YS collected samples. DM and SL designed and conducted the study, and wrote the manuscript.

## Conflict of Interest Statement

The authors declare that the research was conducted in the absence of any commercial or financial relationships that could be construed as a potential conflict of interest.

## Footnotes

1 http://www.ncbi.nlm.nih.gov/blast
