# Supplementary material for: Infection of Goose with Genotype VIId Newcastle Disease Virus of Goose Origin Elicits Strong Immune Responses at Early Stage
Source: Front Microbiol. 2016 Oct 4;7:1587. doi: 10.3389/fmicb.2016.01587 (PMC5047883; doi:10.3389/fmicb.2016.01587)
Supplement: Supplementary file 1 [file Data_Sheet_1.PDF]

Supplementary Material

Infection of goose with genotype VIId Newcastle disease virus of goose origin elicits strong immune responses at early stage

Qianqian Xu, Yuqiu Chen, Wenjun Zhao, Tingting Zhang, Chenggang Liu, Tianming Qi, Zongxi Han, Yuhao Shao, Deying Ma*, Shengwang Liu*

* Corresponding author
Deying Ma
E-mail: madeying@neau.edu.cn

* Corresponding author
Shengwang Liu
E-mail address: swliu@hvri.ac.cn

Supplementary Figure legends

Supplementary Figure 1. Phylogenetic relationships based on the sequences of these anser_AvBDs, other AvBDs, and β-defensins from some mammalian species using the MEGALIGN program DNAStar with Clustal V (Higgins and Sharp, 1988). Sequences identified in the current study are indicated by a star. Accession numbers of the sequences are shown in brackets in the figure. The nucleotide sequences of both anser_AvBD4 and anser_AvBD16 obtained in this study are shown in detail in the **Supplementary Figure** 2. and **Supplementary Figure** 3.

Supplementary Figure 2. The nucleotide sequences of Anser cygnoides avian beta-defensin 4 mRNA.

Supplementary Figure 3. The nucleotide sequences of Anser cygnoides avian beta-defensin 16 mRNA.

Supplementary Figure 4 The nucleotide sequences of Anser cygnoides TLR1 mRNA.

Supplementary Figure 5 The nucleotide sequences of Anser cygnoides Fas ligand mRNA.

Supplementary Figure 6 The nucleotide sequences of Anser cygnoides iNOS mRNA.

Reference

Higgins, D.G. and Sharp, P.M. (1988) CLUSTAL: a package for performing multiple sequence alignment on a microcomputer. *Gene* 73:237-244.

Figure. S1

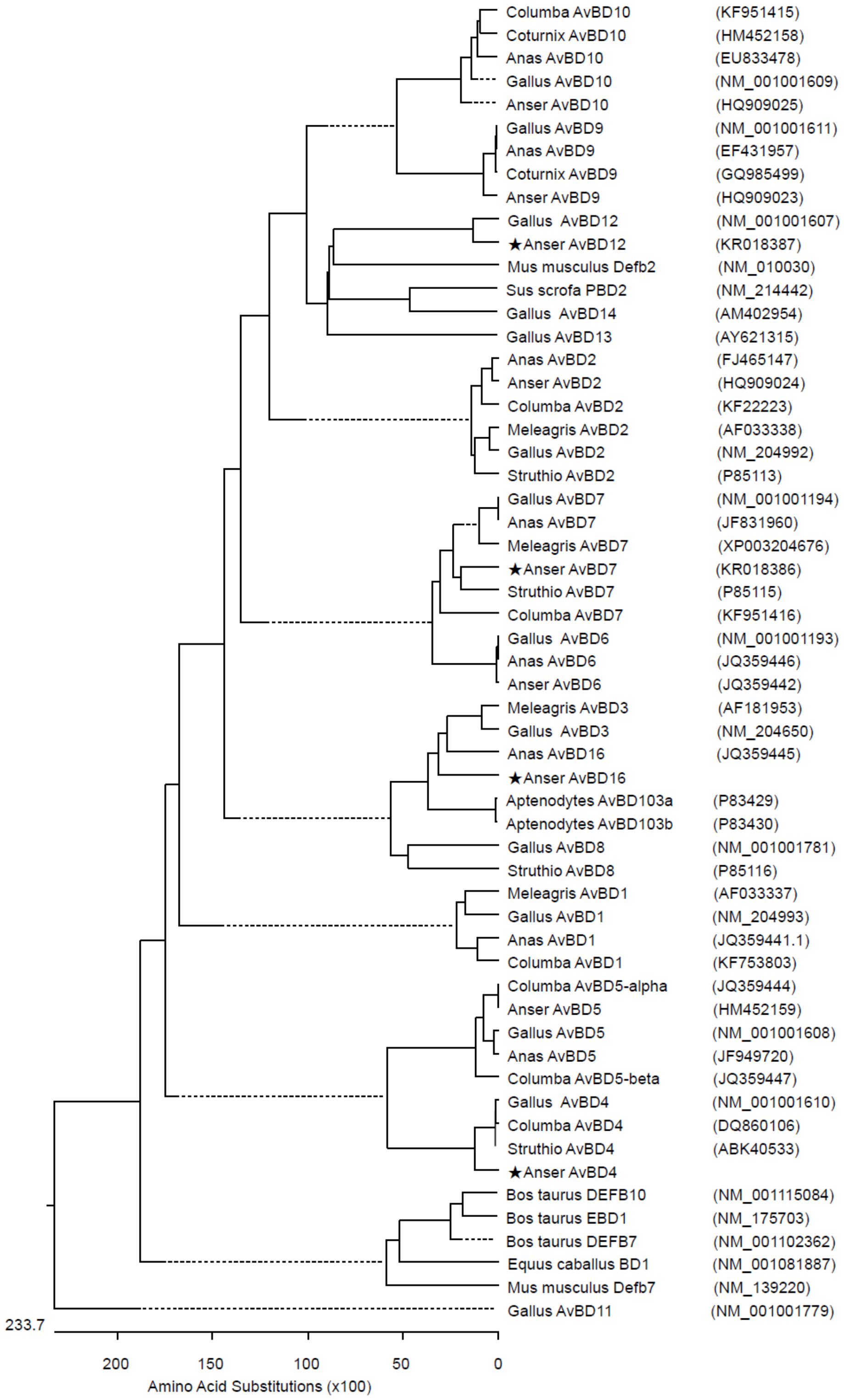

Columba AvBD10 (KF951415)
Coturnix AvBD10 (HM452158)
Anas AvBD10 (EU833478)
Gallus AvBD10 (NM_001001609)
Anser AvBD10 (HQ909025)
Gallus AvBD9 (NM_001001611)
Anas AvBD9 (EF431957)
Coturnix AvBD9 (GQ985499)
Anser AvBD9 (HQ909023)
Gallus AvBD12 (NM_001001607)
★Anser AvBD12 (KR018387)
Mus musculus Defb2 (NM_010030)
Sus scrofa PBD2 (NM_214442)
Gallus AvBD14 (AM402954)
Gallus AvBD13 (AY621315)
Anas AvBD2 (FJ465147)
Anser AvBD2 (HQ909024)
Columba AvBD2 (KF22223)
Meleagris AvBD2 (AF033338)
Gallus AvBD2 (NM_204992)
Struthio AvBD2 (P85113)
Gallus AvBD7 (NM_001001194)
Anas AvBD7 (JF831960)
Meleagris AvBD7 (XP003204676)
★Anser AvBD7 (KR018386)
Struthio AvBD7 (P85115)
Columba AvBD7 (KF951416)
Gallus AvBD6 (NM_001001193)
Anas AvBD6 (JQ359446)
Anser AvBD6 (JQ359442)
Meleagris AvBD3 (AF181953)
Gallus AvBD3 (NM_204650)
Anas AvBD16 (JQ359445)
★Anser AvBD16
Aptenodytes AvBD103a (P83429)
Aptenodytes AvBD103b (P83430)
Gallus AvBD8 (NM_001001781)
Struthio AvBD8 (P85116)
Meleagris AvBD1 (AF033337)
Gallus AvBD1 (NM_204993)
Anas AvBD1 (JQ359441.1)
Columba AvBD1 (KF753803)
Columba AvBD5-alpha (JQ359444)
Anser AvBD5 (HM452159)
Gallus AvBD5 (NM_001001608)
Anas AvBD5 (JF949720)
Columba AvBD5-beta (JQ359447)
Gallus AvBD4 (NM_001001610)
Columba AvBD4 (DQ860106)
Struthio AvBD4 (ABK40533)
★Anser AvBD4
Bos taurus DEFB10 (NM_001115084)
Bos taurus EBD1 (NM_175703)
Bos taurus DEFB7 (NM_001102362)
Equus caballus BD1 (NM_001081887)
Mus musculus Defb7 (NM_139220)
Gallus AvBD11 (NM_001001779)
233.7
200
150
100
50
0
Amino Acid Substitutions (x100)

Figure S2

Anser AvBD4

```
LOCUS       Seq1.                 171 bp    mRNA    linear   VRT 22-MAR-2015
DEFINITION  Anser cygnoides avian beta-defensin 4 mRNA, partial cds.
ACCESSION   Seq1.
VERSION
KEYWORDS    .
SOURCE      Anser cygnoides
  ORGANISM  Anser cygnoides
            Eukaryota; Metazoa; Chordata; Craniata; Vertebrata; Euteleostomi;
            Archelosauria; Archosauria; Dinosauria; Saurischia; Theropoda;
            Coelurosauria; Aves; Neognathae; Galloanserae; Anseriformes;
            Anatidae; Anser.
REFERENCE   1  (bases 1 to 171)
  AUTHORS   Xu,Q., Zhang,T., Ma,D., Liu,S. and Han,Z.
  TITLE     Identification, expression and activity analyses of four novel
            avian beta-defensins from goose
  JOURNAL   Unpublished
REFERENCE   2  (bases 1 to 171)
  AUTHORS   Xu,Q., Zhang,T., Ma,D., Liu,S. and Han,Z.
  TITLE     Direct Submission
  JOURNAL   Submitted (21-MAR-2015) college of animal science and technology,
            Northeast Agricultural University, Wood street no. 59, Harbin, hei
            longjiang 150030, china
COMMENT     Bankit Comment: ALT EMAIL:xuqianqian323@163.com.
            Bankit Comment: TOTAL # OF SEQS:1.
FEATURES             Location/Qualifiers
     source          1..171
                     /organism="Anser cygnoides"
                     /mol_type="mRNA"
                     /db_xref="taxon:8845"
                     /tissue_type="bone marrow"
                     /country="china"
                     /type="mRNA"
     CDS             1..171
                     /codon_start=1
                     /product="avian beta-defensin 4"
                     /translation="-
                     VLLFVAVHGAAGFSRPPKSLMRCGYRGTFCTPGKCPRGNDYLGLCRAEYSCCRWL"
BASE COUNT       26 a     40 c     51 g     54 t
ORIGIN
        1 atcgtgctcc tctttgtggc agttcatgga gctgcaggct tttcccgtcc tccaaagagt
       61 cttatgcgat gtggctatcg tgggaccttc tgcacccctg ggaaatgccc tcgtgggaat
      121 gattatctgg ggctgtgccg tgctgagtat tcttgctgta gatggttgta g//
```

Figure S3

```
Anser AvBD16
LOCUS       Seq1.                 180 bp    mRNA    linear   VRT 22-MAR-2015
DEFINITION  Anser cygnoides avian beta-defensin 16 mRNA, complete cds.
ACCESSION   Seq1.
VERSION
KEYWORDS    .
SOURCE      Anser cygnoides
  ORGANISM  Anser cygnoides
            Eukaryota; Metazoa; Chordata; Craniata; Vertebrata; Euteleostomi;
            Archelosauria; Archosauria; Dinosauria; Saurischia; Theropoda;
            Coelurosauria; Aves; Neognathae; Galloanserae; Anseriformes;
            Anatidae; Anser.
REFERENCE   1  (bases 1 to 180)
  AUTHORS   Xu,Q., Zhang,T., Ma,D., Liu,S. and Han,Z.
  TITLE     Identification, expression and activity analyses of four novel
            avian beta-defensins from goose
  JOURNAL   Unpublished
REFERENCE   2  (bases 1 to 180)
  AUTHORS   Xu,Q., Zhang,T., Ma,D., Liu,S. and Han,Z.
  TITLE     Direct Submission
  JOURNAL   Submitted (22-MAR-2015) college of animal science and technology,
            Northeast Agricultural University, Wood street no. 59, Harbin, hei
            longjiang 150030, china
COMMENT     Bankit Comment: ALT EMAIL:xuqianqian323@163.com.
            Bankit Comment: TOTAL # OF SEQS:1.
FEATURES             Location/Qualifiers
     source          1..180
                     /organism="Anser cygnoides"
                     /mol_type="mRNA"
                     /db_xref="taxon:8845"
                     /tissue_type="bone marrow"
                     /country="China"
     CDS             1..180
                     /codon_start=1
                     /product="avian beta-defensin 16"
                     /translation="MKILCLLFPFFLLFLQGAAGNYGPCGIRGGVCRPGGCRFPERRN
                     GRCSLIVPCCRTVYD"
BASE COUNT     31 a     48 c     50 g     51 t
ORIGIN
        1 atgaagatcc tctgcctgct cttccccttc ttcctcctct ttctccaggg tgctgcagga
       61 aattatggcc cctgtggaat cagaggtgga gtttgtcgtc cagggggctg ccggttccct
      121 gaaaggcgta atgggagatg ttcacttatc gtcccgtgct gcaggactgt gtacgactaa
//
```

Figure S4

Anser TLR1

```
LOCUS       Seq1.                    127 bp    mRNA    linear    VRT 22-MAR-2015
DEFINITION  Anser cygnoides toll-like receptor 1 mRNA, partial cds.
ACCESSION   Seq1.
VERSION
KEYWORDS    .
SOURCE      Anser cygnoides
  ORGANISM  Anser cygnoides
            Eukaryota; Metazoa; Chordata; Craniata; Vertebrata; Euteleostomi;
            Archelosauria; Archosauria; Dinosauria; Saurischia; Theropoda;
            Coelurosauria; Aves; Neognathae; Galloanserae; Anseriformes;
            Anatidae; Anser.
REFERENCE   1  (bases 1 to 127)
  AUTHORS   Xu,Q., Zhang,T., Ma,D., Liu,S. and Han,Z.
  TITLE     Unpublished
  JOURNAL   Unpublished
REFERENCE   2  (bases 1 to 127)
  AUTHORS   Xu,Q., Zhang,T., Ma,D., Liu,S. and Han,Z.
  TITLE     Direct Submission
  JOURNAL   Submitted (22-MAR-2015) college of animal science and technology,
            Northeast Agricultural University, Wood street no. 59, Harbin, hei
            longjiang 150030, china
COMMENT     Bankit Comment: ALT EMAIL:xuqianqian323@163.com.
            Bankit Comment: TOTAL # OF SEQS:1.
FEATURES             Location/Qualifiers
     source          1..127
                     /organism="Anser cygnoides"
                     /mol_type="mRNA"
                     /db_xref="taxon:8845"
                     /tissue_type="spleen"
                     /country="China"
                     /type="mRNA"
     CDS             1..105
                     /codon_start=1
                     /product="toll-like receptor 1"
                     /translation="SPSLCCRPTLCRASGVTMSCTLPITSCLVRIATA"
BASE COUNT      28 a      29 c      30 g      40 t
ORIGIN
        1 agtccatctt tgtgttgtcg cccaactttg tgcagagcga gtggtgtcac tatgagctgt
       61 actttgccca tcacaagctg tttagtgaga attgcaacag cttaatcctg atcttgctgg
      121 agccaat
//
```

Figure S5

Anser FASLG

```
LOCUS       Seq1.                  149 bp    mRNA    linear   VRT 22-MAR-2015
DEFINITION  Anser cygnoides Fas ligand (FASLG), mRNA.
ACCESSION   Seq1.
VERSION
KEYWORDS    .
SOURCE      Anser cygnoides
  ORGANISM  Anser cygnoides
            Eukaryota; Metazoa; Chordata; Craniata; Vertebrata; Euteleostomi;
            Archelosauria; Archosauria; Dinosauria; Saurischia; Theropoda;
            Coelurosauria; Aves; Neognathae; Galloanserae; Anseriformes;
            Anatidae; Anser.
REFERENCE   1  (bases 1 to 149)
  AUTHORS   Xu,Q., Zhang,T., Ma,D., Liu,S. and Han,Z.
  TITLE     Unpublished
  JOURNAL   Unpublished
REFERENCE   2  (bases 1 to 149)
  AUTHORS   Xu,Q., Zhang,T., Ma,D., Liu,S. and Han,Z.
  TITLE     Direct Submission
  JOURNAL   Submitted (22-MAR-2015) college of animal science and technology,
            Northeast Agricultural University, Wood street no. 59, Harbin, hei
            longjiang 150030, china
COMMENT     Bankit Comment: ALT EMAIL:xuqianqian323@163.com.
            Bankit Comment: TOTAL # OF SEQS:1.
FEATURES             Location/Qualifiers
     source          1..149
                     /organism="Anser cygnoides"
                     /mol_type="mRNA"
                     /db_xref="taxon:8845"
                     /country="China"
                     /collection_date="2014"
                     /type="mRNA"
     CDS             3..149
                     /codon_start=1
                     /product="FASLG"
                     /translation="TGNPTQRDLPLEWEPISGHAFTNGIQYRNQGLVINETGLYFVYS
                     NVLFR"
BASE COUNT       32 a     48 c     36 g     33 t
ORIGIN
        1 taacaggaaa ccccacacag cgggacctcc ctttggagtg ggagcccatc tccggccacg
       61 ctttcactaa tggcattcag taccgcaacc agggcctcgt gatcaatgag actggcctgt
      121 acttcgtgta ctccaatgtg ctcttccgg
//
```

48

Figure S6

Anser iNOS

```
LOCUS       Seq1.                         103 bp    mRNA    linear   VRT 14-APR-2015
DEFINITION  Anser cygnoides nitric oxide synthase, mRNA.
ACCESSION   Seq1.
VERSION
KEYWORDS    .
SOURCE      Anser sp.  (goose)
  ORGANISM  Anser sp.
            Eukaryota; Metazoa; Chordata; Craniata; Vertebrata; Euteleostomi;
            Archelosauria; Archosauria; Dinosauria; Saurischia; Theropoda;
            Coelurosauria; Aves; Neognathae; Galloanserae; Anseriformes;
            Anatidae; Anser.
REFERENCE   1  (bases 1 to 103)
  AUTHORS   Xu,Q., Zhang,T., Ma,D., Liu,S.  and Han,Z.
  TITLE     Unpublished
  JOURNAL   Unpublished
REFERENCE   2  (bases 1 to 103)
  AUTHORS   Xu,Q., Zhang,T., Ma,D., Liu,S.  and Han,Z.
  TITLE     Direct Submission
  JOURNAL   Submitted (14-APR-2015) college of animal science and technology,
            Northeast Agricultural University, Wood street no.  59, Harbin, hei
            longjiang 150030, china
COMMENT     Bankit Comment: ALT EMAIL:xuqianqian323@163.com.
            Bankit Comment: TOTAL # OF SEQS:1.
FEATURES             Location/Qualifiers
     source          1..103
                     /organism="Anser sp."
                     /mol_type="mRNA"
                     /db_xref="taxon:8847"
                     /country="China"
                     /collection_date="2013"
                     /type="mRNA"
     CDS             2..103
                     /codon_start=1
                     /product="nitric oxide synthas"
                     /translation="NSQLIRYAGYQMPDGSVVGDPASVEFTKLCIELG"
BASE COUNT     22 a      24 c      34 g      23 t
ORIGIN
        1 gaacagccag ctcatccgat acgctgggta ccaaatgcca gatgggtctg ttgtgggaga
       61 cccggccagc gtggagttca ctaagttgtg cattgagctt ggg
//
```

49
